# Diabetic Muscle Infarction Masquerading as Necrotizing Fasciitis

**DOI:** 10.1155/2017/7240156

**Published:** 2017-04-26

**Authors:** Kalyana C. Janga, Ankur Sinha, Perry Wengrofsky, Phone Oo, Sheldon Greenberg, Regina Tarkovsky, Kavita Sharma

**Affiliations:** ^1^Department of Nephrology, Maimonides Medical Center, Brooklyn, NY, USA; ^2^Department of Medicine, Maimonides Medical Center, Brooklyn, NY, USA; ^3^Technion American Medical Program, Bruce and Ruth Rappaport Faculty of Medicine, Technion-Israel Institute of Technology, Haifa, Israel; ^4^Department of Infectious Disease, Maimonides Medical Center, Brooklyn, NY, USA

## Abstract

A 43-year-old male patient with past medical history of diabetes mellitus (DM), end stage renal disease (ESRD) on hemodialysis (HD), congestive heart failure (CHF), obstructive sleep apnea (OSA), and chronic anemia presented with complaints of left thigh pain. A computerized tomogram (CT) of the thigh revealed evidence of edema with no evidence of a focal collection or gas formation noted. The patient's clinical symptoms persisted and he underwent magnetic resonance imaging (MRI) of his thigh which was reported to show small areas of muscle necrosis with fluid collection. These findings in the acute setting concerned necrotizing fasciitis. After careful discussion following a multidisciplinary approach, a decision was made to perform a fasciotomy with tissue debridement. The patient was treated with IV antibiotics and discharged with a vacuum assisted wound drain. The surgical pathology revealed evidence of muscle edema with necrosis. Seven weeks later the patient presented with similar complaints on the other thigh (right thigh). MRI of the thighs revealed worsening edema with features suggestive of myositis and possible muscle infarction. A CT guided biopsy of the right quadriceps muscle revealed fibrotic interstitial connective tissue and no evidence of necrosis. This favored a diagnosis of diabetic muscle infarction. The disease was managed with pain control, strict diabetes management, and aggressive dialysis.

## 1. Introduction

Diabetic muscle infarction (DMI) is a rare complication of diabetes mellitus and has been scarcely reported in medical literature [1]. This condition usually involves the lower limbs and affects individuals with a long-standing history of diabetes. It is associated with other complications of persistent hyperglycemia like neuropathy, retinopathy, and nephropathy [2]. The presenting symptoms of acute debilitating pain, with swelling of the affected muscles, make it a difficult diagnosis. This is due to a significant overlap of clinical features with other etiologies. We report a patient who presented with features of DMI not distinguishable from necrotizing fasciitis (NF); the patient underwent emergent surgical debridement leading to significant morbidity.

## 2. Case Presentation

A 43-year-old male patient with past medical history of diabetes mellitus (DM), end stage renal disease (ESRD) on hemodialysis (HD), congestive heart failure (CHF), obstructive sleep apnea (OSA), and chronic anemia presented with complaints of left thigh pain. The pain was constant and sharp; it worsened with movement and localized to the medial aspect of the left thigh. The patient denied any fever, chills, or any localized trauma to the area. Physical examination revealed marked tenderness to the left thigh with no evidence of a skin rash or edema. Blood work was within normal limits apart from an elevated creatine phosphokinase (CPK) level of 533 IU/L (normal range 30–135 IU/L) and an elevated serum myoglobin of 494 ng/ml (normal range 7.0–46.2 ng/mL). Blood was collected for culture.

**Figure 1:**
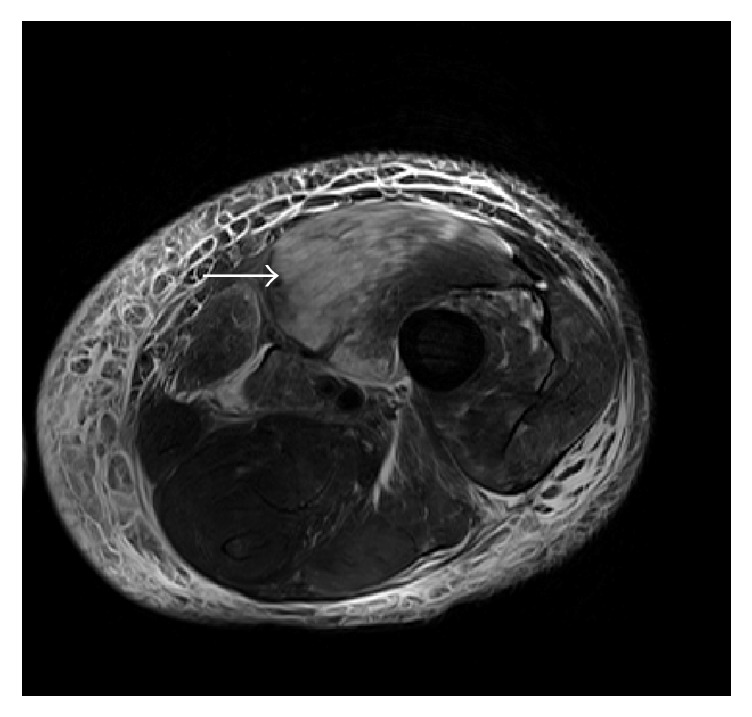
MRI of the left thigh, coronal section, showing hyperintense signals on T2 weighted images (white arrow).

**Figure 2:**
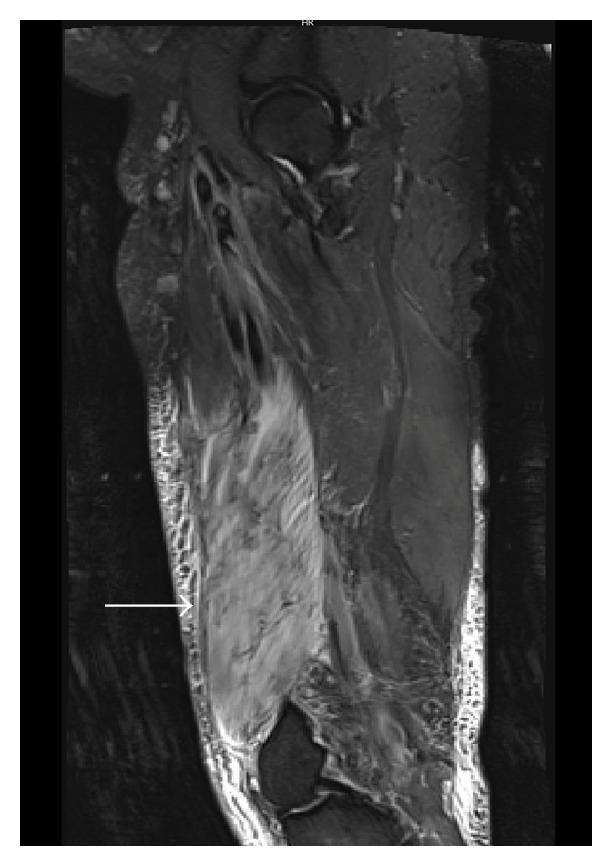
MRI of the left thigh, sagittal section, showing hyperintense signals on T2 weighted images (white arrow).

**Figure 3:**
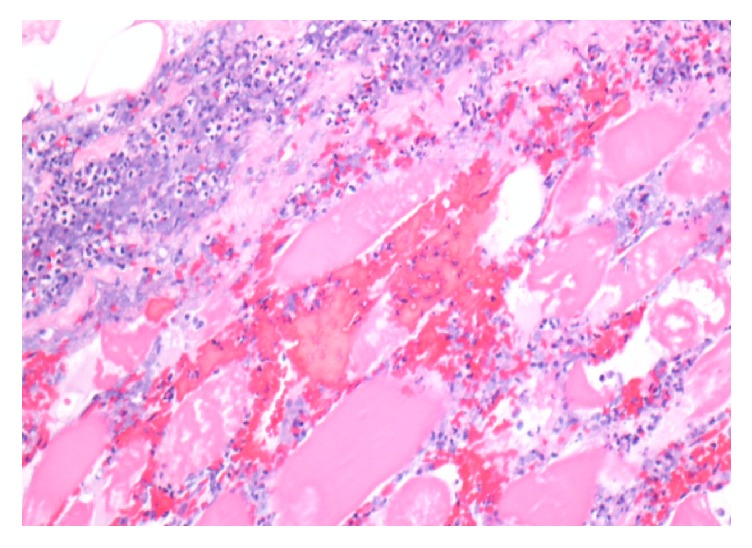
Histological section of the muscle biopsy, showing intense neutrophilic infiltration with evidence of skeletal muscle edema and necrosis.

A computerized tomogram (CT) of the thigh revealed evidence of edema with no evidence of a focal collection or gas formation noted. The patient's clinical symptoms persisted and he underwent magnetic resonance imaging (MRI) of his thigh, which was reported to show small areas of muscle necrosis with fluid collection (Figures 1 and 2). These findings in the acute setting concerned necrotizing fasciitis. After careful discussion following a multidisciplinary approach, a decision was made to perform a fasciotomy with tissue debridement. The patient was treated with IV antibiotics and discharged with a vacuum assisted wound drain. The surgical pathology (Figure 3) revealed intense neutrophilic infiltration with evidence of skeletal muscle edema with adjoining necrosis. These findings were nonspecific and favored an infectious process.

**Figure 4:**
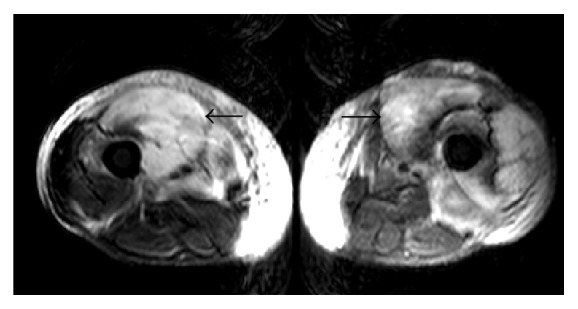
MRI of the thighs, coronal section, showing hyperintense signals on T2 weighted images bilaterally (black arrows).

Seven weeks later, the patient presented with similar complaints on the other thigh (right thigh). The pain was similar to the previous presentation and the patient reported that the pain in his left thigh had persisted despite surgical debridement. Blood work remained unremarkable apart from an elevated creatinine phosphokinase (CPK) level of 696 IU/L (normal range 30–135 IU/L) and an elevated serum myoglobin of 1702 ng/ml (normal range 7.0–46.2 ng/mL). CT scan of the right thigh revealed marked subcutaneous edema with linear fluid collections. MRI of the thighs revealed worsening edema with features suggestive of myositis and possible muscle infarction (Figure 4). A CT guided biopsy of the right quadriceps muscle revealed fibrotic interstitial connective tissue and no evidence of necrosis. The pathological features, with bilateral involvement and repeat episodes, favored a diagnosis of diabetic muscle infarction. The disease was managed with pain control, strict diabetes management, and aggressive dialysis.

## 3. Discussion

DMI is a clinical entity that is rarely reported and usually involves patients with poorly controlled diabetes. The basic pathology is nonenzymatic glycosylation of proteins leading to microangiopathic complications [3]. This is supported by the fact that most of the patients suffering from DMI already have other concomitant microvascular diseases [4]. DMI is characterized by sudden pain and swelling of the affected muscle. Muscles of the thigh are most frequently affected [2]. Usually, there are no signs of systemic infection, like fever or chills. Laboratory tests can be within normal range, usually revealing normal white cell count, erythrocyte sedimentation rate, normal or elevated inflammatory markers including erythrocyte sedimentation rate, and normal to mildly elevated CPK [5].

Diagnosis of DMI remains a challenge. Various imaging modalities have been discussed in literature but MRI has become the modality of choice [2]. MRI findings include hyperintense signals on T2 weighted images and isointense to hypointense signals on T1 weighted images [6]. Ultrasound shows marginated, heterogeneous, mass-like echogenic changes with loss of myofascial interfaces [7]. These findings are nonspecific and depict muscle swelling. CT scan of the affected muscle shows increased muscle size and higher attenuation [6].

Muscle biopsy can provide a definitive diagnosis. The biopsy demonstrates areas of infarction with coagulative necrosis. There is evidence of microangiopathy with vessel and endothelial swelling. There can be neutrophilic infiltration and edema. Late histologic features include advanced muscle fiber fibrosis, atrophy, and muscle fiber regeneration with lymphocytic infiltration [2, 8]. Biopsy with minimally invasive approach is preferred over open surgical biopsy [1].

The differential diagnosis of DMI includes infectious as well as vascular etiologies. In our patient the clinical evidence pointed towards cellulitis with possible necrotizing fasciitis, and the significant morbidity associated with delay in surgical debridement was pivotal in our inclination towards a surgical approach.

Management of DMI mainly consists of supportive care with analgesics, limitation of activity, and strict glycemic control in the acute phase of the disease [9]. Very rarely surgical resection can be warranted for symptomatic relief [1]. There is a high risk of recurrence and therapy should be aimed at minimizing hospital stay and reducing recovery times. This can be achieved with adequate glycemic control [2]. Medical management also includes aggressive and prolonged dialysis to achieve adequate dry weight, raising urea clearance and high flux, and high efficiency dialyzers.

Patients with ESRD are at a higher risk of acquiring infections than the general population, with infection being the second highest cause of mortality in patients with ESRD [10]. Uremia in ESRD causes secondary immune failure by multiple pathogenic mechanisms, including lymphocyte dysfunction, impaired production of IL-2 and other interleukins, enhanced monocytic production of proinflammatory cytokines, and diminished phagocytosis [11, 12]. Nonimmune system factors predisposing ESRD patients to infection include invasive dialysis procedures, malnutrition, disruption of skin and mucosal barriers, and susceptibility to nosocomial transmission [9].

Based on the experience with this patient, the similarities in presentation between DMI and NF, the overlap of risk factors for both conditions, and the overwhelming difficulty in distinguishing the two, we encourage a highly coordinated multidisciplinary approach. Appropriate steps should be taken to minimize hospital stay and to avoid exposure to nosocomial infections.
